# Sepsis Definitions: The Search for Gold and What CMS Got Wrong

**DOI:** 10.5811/westjem.2017.4.32795

**Published:** 2017-07-10

**Authors:** Annahieta Kalantari, Haney Mallemat, Scott D. Weingart

**Affiliations:** * Aria Jefferson Health, Department of Emergency Medicine, Philadelphia, Pennsylvania; † Philadelphia College of Osteopathic Medicine, Department of Emergency Medicine, Philadelphia, Pennsylvania; ‡ Cooper Medical School of Rowan University, Department of Emergency Medicine, Department of Critical Care Medicine, Camden, New Jersey; § Stony Brook Medicine, Department of Emergency Medicine, Division of Emergency Critical Care, Stony Brook, New York

## Abstract

On October 1, 2015, the United States Centers for Medicare and Medicaid Services (CMS) issued a core measure addressing the care of septic patients. These core measures are controversial among healthcare providers. This article will address that there is no gold standard definition for sepsis, severe sepsis or septic shock and the CMS-assigned definitions for severe sepsis and septic shock are premature and inconsistent with evidence-based definitions.

## INTRODUCTION

The Centers for Medicare and Medicaid Services (CMS) issued core measures for the management of sepsis on October 1, 2015, which state that “the evidence cited for all components of this measure is directly related to decreases in organ failure, overall reductions in hospital mortality, length of stay, and costs of care.”^1^ This is an admirable statement but may not be the case when these core measures are applied at bedside mainly because statements within the measure are not fully supported with evidence-based literature. These problems start at the very beginning with the CMS-designated definitions of severe sepsis and septic shock.

Since 1992, the definitions of sepsis, severe sepsis and septic shock have been heavily debated. Multiple consensus statements have been released.^2^–^4^ Each iteration has attempted to incorporate concepts reflecting an updated understanding of the pathophysiology of sepsis. Yet none have been perfect or accepted as gold standard.^2^–^9^

CMS neglected to acknowledge that there is no perfect definition available for sepsis, severe sepsis and septic shock and it is premature to institute government-mandated sepsis core measures. Additionally, the definitions they selected are inconsistent with the definitions used in evidence-based studies since 2001.The problems continue as these imperfect and inconsistent mandatory definitions serve as the trigger to a cascade of resuscitative efforts. To add to the dilemma, if a clinician is noncompliant with *any* portion of this measure, hospital reimbursement is withheld.

The CMS-proposed definitions are a deviation from the definitions that clinicians have used in their medical practice for nearly 15 years. The major difference is with the value of lactate and whether fluid resuscitation has occurred. We detail the history of the definitions in the sepsis syndrome continuum from their inception to present day and demonstrate that the CMS-proposed definitions are not supported by evidence and should not be used as a trigger to initiate the rest of the CMS resuscitation cascade.

## CASE

A 55-year-old, morbidly obese male presents to the emergency department (ED) with a chief complaint of severe abdominal pain. The pain started approximately two days ago and he also reports anorexia, nausea and dysuria. His vitals signs are T 101.5°F, BP 134/68, HR 110, RR 20, pulse oximetry 98% on room air, weight 138 kilograms. On physical exam, he has dry mucous membranes, is tachycardic, and has diffuse lower abdominal pain. Basic labs are drawn, an intravenous line is started and crystalloid fluids are given at a rate of wide open.

A leukocytosis of 23,000 without a bandemia and a lactate of 4 mmol/L was found on review of his labs. Urine analysis reveals presence of a urinary tract infection; the rest of his lab tests are unremarkable. Appropriate antibiotics are started. The question now: Is your patient septic, severely septic or in septic shock?

## A HISTORY LESSON

The American College of Chest Physicians (ACCP) and Society for Critical Care Medicine (SCCM) released a consensus statement in 1992 that provided the first published definitions for systemic inflammatory response syndrome (SIRS), sepsis, severe sepsis, septic shock, sepsis-induced hypotension and multiple organ dysfunction syndrome (MODS). The consensus statement provided robust verbal definitions and assigned objective clinical criteria for SIRS criteria, but did not supply specific clinical criteria to define end-organ dysfunction.^2^ These definitions are provided in Table 1.

A study by Sands et al. in 1997 used strict criteria to define the epidemiology of the sepsis syndrome, which was defined as the presence of either temperature > 38.2°C or < 35.6°C measured rectally, respirations > 20 breaths per minute or the need for mechanical ventilation, heart rate > 90 beats per minute AND clinical evidence of infection OR one or more blood cultures positive for a pathogen at 48 hours. Additionally, the study provided the first clinical criteria used to define severe sepsis and septic shock, which included any one of the following: 1) PaO2/FiO2 < 280, arterial pH < 7.30; 2) urine output < 30mL/h; 3) systolic blood pressure (SBP) < 90 mm Hg or fall in SBP > 40 mm Hg sustained for two hours despite fluid challenge; 4) systemic vascular resistance < 800 dynes/s/cm; 5) prothrombin time or partial thromboplastin time > normal; or 6) platelets < 100.0 × 10^9/L or platelets decreased to < 50% of most recent measurement before current day; or 7) documentation of deterioration in mental status within 24 hours.^10^

Emmanuel Rivers’ landmark sepsis trial in 2001 cited both the ACCP/SCCM consensus definitions ***and*** the Sands study definitions for sepsis, severe sepsis and septic shock. In the Rivers’ trial, patients were included when two of four SIRS criteria were present and a SBP of no higher than 90 mm Hg after crystalloid fluid challenge or the patient had a blood lactate concentration of 4 mmol per liter or greater.^11^ Many subsequent studies have evaluated patients with severe sepsis and septic shock using these Rivers’ definitions.

In 2003 Levy et al. published an article in *Intensive Care Medicine* that detailed the 2001 SCCM/ESICM/ACCP/ATS/SIS International Sepsis Definitions. This publication introduced updated concepts in sepsis pathophysiology and clinical data to expand the definitions first published in 1992. It is in these definitions that hyperlactatemia, defined as > 3mmol/L, is first mentioned as diagnostic criteria for sepsis. It is also in this publication that the authors stated, “*Unfortunately, a clinically useful set of criteria for diagnosing sepsis and related conditions will necessarily be somewhat arbitrary. There is no ‘gold standard’ against which the diagnostic criteria can be calibrated*.” 3

In 2004 the Surviving Sepsis Campaign released its initial guidelines for sepsis management in the journals of *Critical Care Medicine* and *Intensive Care Medicine*. Since that time, three additional editions of the Surviving Sepsis Campaign Guidelines (SSCG) have been published with the most recent being in 2016.^5^–^8^ The 2012 definitions are the last to include specific clinical criteria to identify sepsis. Within the article were two conflicting values for an abnormal lactate level. In one table, a lactate > 1 mmol/L defined hyperlactatemia and the Levy article was cited for this value. In a separate table, “*Lactate above upper limits of laboratory normal*” was listed as evidence of end-organ dysfunction.^7^

In 2012 the National Quality Forum published definitions and quality measures for the management of sepsis, severe sepsis and septic shock. The definitions and bundles of this measure were the 2012 SCC guidelines verbatim.^12^

Between 2014 and 2015, three separate randomized controlled trials were published (PROCESS, ARISE, and PROMISE) that evaluated the mortality of patients receiving early goal-directed therapy (EGDT) versus usual care. The PROCESS study recruited ED patients who on presentation had two or more SIRS criteria, lactate > 4mmol/L and who had refractory hypotension as a SBP that either was less than 90 mm Hg or required vasopressor therapy to maintain 90 mm Hg despite an intravenous (IV) fluid challenge of crystalloid. A fluid challenge was defined as 20 ml or more per kilogram of body weight, administered over 30 minutes at the beginning of the study, but was later simplified to 1,000 ml or more administered over 30 minutes.^13^ The ARISE trial investigators included patients with a suspected or confirmed infection, two or more SIRS criteria, and evidence of refractory hypotension or hypoperfusion (defined as lactate > 4 mmol/L). Refractory hypotension was defined as a SBP < 90 mm Hg or a mean arterial pressure (MAP) < 65 mmHg after an IV fluid challenge of 1,000 ml or more of crystalloid administered over a 60-minute period.^14^ The PROMISE trial investigators enrolled patients with a known or presumed infection, two or more SIRS criteria and either refractory hypotension (i.e., the same definition as the ARISE trial) or hyperlactatemia (lactate > 4 mmol/L).^15^

In 2016 Singer and authors released the Sepsis-3 consensus paper, which eliminated severe sepsis entirely and changed the definitions for sepsis and septic shock. The Sepsis-3 definition of sepsis is a “*life-threatening organ dysfunction cause by a dysregulated host response to infection.”* Clinically this is detected by suspected or documented infection and two or more quick Sequential Organ Failure Assessment (qSOFA criteria (Table 2). Septic shock is a “*subset of sepsis in which underlying circulatory and cellular/metabolic abnormalities are profound enough to substantially increase mortality.*” Clinically this is detected in the setting of sepsis and vasopressor therapy needed to elevated MAP ≥ 65 mm Hg AND a lactate > 2 mmol/L despite adequate fluid resuscitation. The authors highlight those concerns addressed in the Levy paper by saying, “*sepsis is a broad term applied to an incompletely understood process. There are, as yet, no simple and unambiguous clinical criteria or biological, imaging, or laboratory features that uniquely identify a septic patient.”*^4^

The most recent Surviving Sepsis Campaign guidelines were released in early 2017. Unlike the previous releases, this version accepted some of the Sepsis-3 proposals and eliminated severe sepsis as a category. SSC also accepted the proposed verbal definitions for sepsis and septic shock. However, qSOFA was not accepted or recommended as best practice, and SIRS along with all other specific clinical parameters of end-organ dysfunction were eliminated from the recommendations.^8^

## WHAT ARE THE CMS DEFINITIONS?

The CMS sepsis core measures detail different clinical criteria and parameters that define the qualifications for severe sepsis and septic shock. The CMS definition of severe sepsis is an infection or suspected infection with two or more SIRS criteria plus one sign of organ dysfunction (Table 3).

The definition of septic shock is a patient with either 1) SBP < 90 mm Hg, 2) a mean arterial pressure < 65 mm HG, or 3) a reduction in SBP by more than 40 mm Hg from a previously recorded measurement (e.g., in a clinic visit). These criteria are valid only after the patient has received a 30 mL/kg crystalloid fluid bolus or with the initial lactate level greater than or equal to 4 mmol/L.^1^ Table 4 illustrates the evolving and proposed definitions for sepsis, severe sepsis and septic shock.

## SO WHAT’S WRONG WITH THE CMS DEFINITIONS?

There are two main problems with the CMS proposed definitions. First, the CMS definition-selected lactate values are below the threshold of widely accepted and studied lactate levels. The second is the very existence of government-issued definitions for a disease state that presents with a great deal of variability and where no gold standard definitions exist.

The CMS definitions are derived from the SCC and NQF definitions, but CMS definitions independently altered the threshold values for lactate. According to CMS, a lactate > 2 mmol/L now represents a patient with severe sepsis and an initial lactate > 4 mmol/L defines a patient in septic shock. You will recall that prior studies used a lactate cutoff of greater than 4mmol/L to define severe sepsis. It was only if the lactate level remained elevated after a fluid resuscitation were patients categorized as being in septic shock. The derivation of these specific lactate values and the proposed values included in the CMS definitions is unknown because CMS does not reference the source of these values.

Studies have demonstrated a distinct leap in mortality rates of septic patients presenting with a lactate level > 4mmol/L.^11^,^16^–^20^ Mikkelson et al. demonstrated that an elevated lactate is an independent predictor of mortality. In their study, they evaluated the significance of intermediate lactate levels (2–3.9 mmol/L) and found a two-fold increase in mortality when compared to severely septic patients with values less than 2 mmol/L.^18^

Other studies have also demonstrated increased mortality rates in intermediate lactate groups,^17^,^20^ but did not evaluate the benefit of aggressive resuscitation in these patients. One study conducted by Liu et al. demonstrated improved mortalities after initiation of aggressive resuscitative measures in patients with intermediate lactate levels.^21^

Yet many other studies have illustrated the negative effects of overly aggressive resuscitation in septic, severely septic and septic shock patients.^22^–^26^

In changing the clinically significant value of lactate, CMS mandated that clinical practice, hospital protocols, and medical education had to adopt the lower threshold of 2 mmol/L to define severe sepsis and an initial lactate of greater than 4 mmol/L to define septic shock in the absence of robust supportive literature. Physicians are being forced to use government-issued standards of practice and patient care that have not been fully investigated as appropriate and safe. Doctors are no longer permitted to doctor but rather forced to practice cookie cutter one-size-fits-all algorithms with regard to sepsis care. These constraints leave the clinician in the predicament of using best practices versus following mandated guidelines.

We have demonstrated that there are various proposed definitions for sepsis, severe sepsis and septic shock. This is likely due to the fact that unlike myocardial infarction, which has a very precise pathophysiology and organic effect, sepsis is a spectrum of any number of factors. It is not due to one distinct insult but can be caused by a large variety of infectious agents that can infect a variety of anatomic locations. It is not due to one region of the body suffering hypoxia; rather it is due to a dysregulated host response to infection. And that host response is dependent on a variety of uncontrolled factors such as age, sex and comorbidities. It may be impossible to develop definitions that appropriately identify a disease state that is so dependent on multiple variables. Each patient is different and cannot be defined and treated exactly the same way. The CMS definitions are premature and, unlike the various other definitions presented, are mandatory and must be followed by clinicians practicing in the United States.

## BACK TO OUR CASE

Is your patient septic, severely septic or in septic shock? The answer is dependent on the set of definitions being used.

Using the CMS definition, the patient above is in septic shock and requires a 30 mL/kg bolus of fluids, which translates to a mandated 4,140 milliliters of fluid bolus, a perfusion reassessment physical exam, repeat lactate and vasopressors if the patient develops hypotension. Based on 2012 SSC guidelines, the patient is severely septic and is suggested to receive a 30 mL/kg bolus of fluid and have a repeat lactate drawn. Based on the Sepsis-3 definitions, the patient is neither septic nor in septic shock and no treatment cascade exists as these are a consensus statement and not treatment guidelines. This patient meets three vastly different definitions, a quandary that highlights the variability of existing definitions. It also highlights the differences between government-mandated definitions versus recommendations versus consensus papers. Mortality rates differ among patients with sepsis, severe sepsis and septic shock. This is the same patient who can have a mortality rate from 4% to 40% depending on which definition is used. Lastly, these vastly different definitions influence the disposition of the patient. Regardless of the set of definitions under use, the majority of clinicians recognize that this patient requires IV antibiotics, fluid resuscitation and hospital admission. Unfortunately for this patient, the hospital reimbursement is based solely on compliance with the CMS core measures and administration of just over four liters of fluids and not a physician’s clinical acumen.

## CONCLUSION

The field of medicine is fluid and dynamic. The practices of today are vastly different from 20 years ago and will be different in 20 years from now. But these changes that our field undergoes are based on evidence and science. Government-issued and -mandated health policy incongruent with evidence-based medicine is detrimental and counterproductive to patient care. If the goal is indeed to achieve “decreases in organ failure, overall reductions in hospital mortality, length of stay, and costs of care” then the core measures must be backed by evidence-based medicine. It is premature to assign mandated definitions to a complex disease spectrum. It is premature to lower lactate thresholds without the backing of robust studies to demonstrate the safety of aggressive resuscitation in these patients. These definitions are a weak start to a broken healthcare policy.

## Figures and Tables

**Table 1:** Adapted from ACCP/SCCM consensus statement.

	Definition
SIRS	
Criteria	Two or more of the following
	Temperature > 38°C or < 36°C
	Heart rate > 90 beats per minute
	Respiratory rate > 20 breaths per minute or PaCO2 < 32 mm Hg
	White blood cell count > 12,000/cu mm, < 4,000/cu mm or
	> 10% immature (band) forms
Sepsis	The systemic response to infection manifested by 2 or more SIRS criteria
Severe sepsis	Sepsis associated with organ dysfunction, hypoperfusion or hypotension that may include but are not limited to, lactic acidosis, oliguria or an acute alteration in mental status
Septic shock	Sepsis-induced with hypotension despite adequate fluid resuscitation along with the presence of perfusion abnormalities that may include, but are not limited to, lactic acidosis, oliguria, or an acute alteration in mental status. Patients who are receiving inotropic or vasopressor agents may not be hypotensive at the time that perfusion abnormalities are measured.
Sepsis-induced hypotension	A systolic blood pressure < 90 mm Hg or a reduction of >/− 40 mm Hg from baseline in the absence of other causes for hypotension
MODS	Presence of altered organ function in acutely ill patients such that homeostasis cannot be maintained without intervention

*ACCP*, American College of Chest Physicians; *SCCM*, Society of Critical Care Medicine; *SIRS*, systemic inflammatory response syndrome; *PaCO2*, partial pressure of carbon dioxide in arterial blood; *MODS*, multiple organ dysfunction syndrome.

**Table 2:** Quick Sequential Organ Failure Assessment criteria.

Altered mental status
Systolic blood pressure < 90 mm Hg
Respiratory rate ≥ 22 breaths per minute

**Table 3:** CMS evidence of organ dysfunction.

Lactate > 2 mmol/L
INR > 1.5 or aPTT > 60 seconds
Platelet count < 100,000 μL^−1^
Bilirubin > 2mg/dL
Creatinine > 2 mg/dL
Urine output < 0.5mL/kg/hour × 2 hours
Acute respiratory failure by need for new invasive or noninvasive ventilation.
Systolic blood pressure < 90 mm Hg or MAP < 65 mm Hg or decreased in SBP more than 40 mm Hg from previously recorded patient normal.

*CMS*, Centers for Medicare and Medicaid Services; *INR*, international normalized ratio; *aPTT*, activated partial thromboplastin time, *MAP*, mean arterial pressure; *SBP*, systolic blood pressure.

**Table 4:** Evolution of sepsis, severe sepsis and septic shock definitions with clinical criteria.

	1992 ACCP/SCCM Consensus statement	Levy	2012 SCCG	NQF	CMS	Sepsis-3	2016 SCCG
SIRS	Temperature > 38°C or < 36°C Heart rate > 90 bpm Respiratory rate > 20 or PaCO2 < 32 mm Hg White blood cell count > 12,000/cu mm, <4,000/cu mm or >10% bands	No change	No change	No change	No change	Eliminated and qSOFA introduced for purpose of risk stratification	No SIRS. No qSOFA.
Sepsis	Infection + 2 or more SIRS	No change	No change	No change	No change	Infection + 2 qSOFA criteria	Infection + end organ dysfunction. No clinical criteria offered.
Severe sepsis	Sepsis + end-organ dysfunction. No specific lactate level offered.	Sepsis + end-organ dysfunction. Lactate > 3*	Sepsis + end-organ dysfunction. Lactate > 4	No change	Sepsis + end-organ dysfunction. Lactate > 2	Eliminated	Eliminated
Septic shock	Sepsis + a SBP <90 mm Hg or a reduction of 40 mm Hg from baseline or evidence of low perfusion after adequate fluid bolus. No specific lactate level offered.	Same as 1992 with addition of MAP < 60 mm Hg despite adequate fluid bolus.	MAP threshold increased to < 70 mm Hg and fluid bolus defined as 30 mL/kg	No change	Initial lactate > 4 or SBP < 90 mm Hg after 30 mL/kg fluid bolus	SBP < 90 mm Hg AND lactate > 2 after adequate fluid resuscitation	Subset of sepsis with circulatory and cellular/metabolic dysfunction associated with a higher risk of mortality. No clinical criteria offered.

*MAP*, mean arterial pressure, *SBP*, systolic blood pressure.

* all lactate levels in mmol/L values.
